# To gate or not to gate - dosimetric evaluation comparing Gated vs. ITV-based methodologies in stereotactic ablative body radiotherapy (SABR) treatment of lung cancer

**DOI:** 10.1186/s13014-016-0699-2

**Published:** 2016-09-22

**Authors:** Joshua Kim, Qixue Wu, Bo Zhao, Ning Wen, Munther Ajlouni, Benjamin Movsas, Indrin J. Chetty

**Affiliations:** Department of Radiation Oncology, Henry Ford Health System, 2799 W. Grand Blvd, Detroit, MI 48202 USA

**Keywords:** SABR, Gating, ITV-based planning, Treatment planning

## Abstract

**Background:**

To compare retrospectively generated gated plans to conventional internal target volume (ITV)-based plans and to evaluate whether gated radiotherapy provides clinically relevant dosimetric improvements to organs-at-risk (OARs).

**Methods:**

Evaluation was performed of 150 stereotactic ablative radiotherapy treatment plans delivered to 128 early-stage (T1-T3 (<5 cm)) NSCLC patients. To generate gated plans, original ITV-based plans were re-optimized and re-calculated on the end-exhale phase and using gated planning target volumes (PTV). Gated and ITV-based plans were produced for 3 × 18 Gy and 4 × 12 Gy fractionation regimens. Dose differences between gated and ITV-based plans were analyzed as a function of both three-dimensional motion and tumor volume. OARs were analyzed using RTOG and AAPM dose constraints.

**Results:**

Differences between gated and ITV-based plans for all OAR indices were largest for the 3 × 18 Gy regimen. For this regimen, MLD differences calculated by subtracting the gated values from the ITV-based values (ITV vs. Gated) were 0.10 ± 0.56 Gy for peripheral island (*N* = 57), 0.16 ± 0.64 Gy for peripheral lung-wall seated (*N* = 57), and 0.10 ± 0.64 Gy for central tumors (*N* = 36). Variations in V20 were similarly low, with the greatest differences occurring in peripheral tumors (0.20 ± 1.17 %). Additionally, average differences (in 2Gy-equivalence) between ITV and gated lung indices fell well below clinical tolerance values for all fractionation regimens, with no clinically meaningful differences observed from the 4 × 12 Gy regimen and rarely for the 3 × 18 Gy regimen (<2 % of cases). Dosimetric differences between gated and ITV-based methods did generally increase with increasing tumor motion and decreasing tumor volume. Dose to ribs and bronchial tree were slightly higher in gated plans compared to ITV-based plans and slightly lower for esophagus, heart, spinal cord, and trachea.

**Conclusions:**

Analysis of 150 SABR-based lung cancer treatment plans did not show a substantial benefit for the gating regimen when compared to ITV-based treatment plans. Small benefits were observed only for the largest tumor motion (exceeding 2 cm) and the high dose treatment regimen (3 × 18 Gy), though these benefits did not appear to be clinically relevant.

## Background

Stereotactic ablative radiotherapy (SABR), also called stereotactic body radiation therapy, is a radiotherapy treatment method for delivering high dose in few fractions (~1–5) [1]. It is essential that the high doses used in SABR treatments be highly conformal to the tumor volume and delivered with high accuracy. For lung cancer, SABR has been used as the primary treatment in prospective trials for medically inoperable, early stage non-small-cell lung cancers (NSCLC) [2] and has provided much greater local tumor control after three years relative to conventionally fractionated radiotherapy (~90 % [3, 4] vs. <55 % [5]) leading to a higher overall 3-year survival rate (~56 % [4] vs. 20–40 % [5, 6]).

Due to respiratory motion of the tumor, immobilization and motion management for lung cancer is important for accurately defining and treating the tumor. The immobilization devices used are somewhat dependent on the motion management strategy used. They come in many forms including cradles that hold a polyurethane foam that conforms to the patient’s body as it cures [7], cushions that conform to the patient’s contours and become rigid as air is evacuated (e.g. BodyFIX®) [7, 8], and abdominal compression plates that constrict abdominal motion [9]. Several types of strategies have been introduced to mitigate the effects of motion. These techniques include: motion encompassing methods, respiratory gating, breath-hold control, forced compression approaches, and tumor-tracking [10]. Many of these methods employ four-dimensional computed tomography (4DCT), where the patients’ respiratory waveforms are monitored during very low pitch helical CT acquisitions [11–13]. These waveforms are then used to retrospectively sort the projections into a predetermined number of phases of the breathing cycle, and the binned projections are used to reconstruct a CT of the patient at each phase. These phase images can be further used to generate derived images such as the average, maximum intensity, and minimum intensity image sets. One type of motion-encompassing method involves the use of 4DCT phase and derived images to define the full range of motion during respiration and to include that full range within an internal target volume (ITV) [14] (or alternatively using a mid-ventilation approach with a MidV volume [15]), which is then expanded to the planning target volume (PTV) for treatment planning. Treatment planning is then typically performed on the average CT. Respiratory gated radiotherapy involves monitoring an internal [16] (e.g. implanted fiducial markers) or external [17] (e.g. abdominal surface motion) surrogate for the tumor. The beam is activated only when the surrogate lies within some predefined gating window on the waveform. This enables the use of the reconstructed image of only a single phase of the waveform and the use of smaller treatment planning margins that are expected to allow for reduced dose delivered to surrounding organs-at-risk (OARs). In tumor tracking modalities, real-time image guidance is employed to dynamically modify the beam delivery so that the radiation delivered tracks with the motion of the target. This can be accomplished through moving the linac itself through use of a robotic arm [18] or using the multi-leaf collimators (MLCs) to track with the tumor [19]. While the previous methods allow for the patient to breath normally, breath-hold methods seek to limit the tumor motion by only treating during the time patients are holding their breath [20–22]. Forced compression methods mechanically limit the range of motion by using a plate to physically compress the abdomen during delivery [23]. Patients with inoperable lung tumors already suffer from compromised breathing and often suffer from comorbidities, and, therefore, only a subset are able to hold their breath for sufficiently long times for breath-hold methods. Similarly, some patients are not able to handle forced compression of their abdomen, and accurate repositioning of the abdominal compression plate is sometimes difficult.

The American Association of Physicists in Medicine (AAPM) Task Group 76 [10] recommends use of a 5 mm motion threshold above which motion management is needed. In this study, respiratory gated treatment plans were retrospectively generated for a large cohort of SABR treatment plans (*n* = 150) that had been planned and treated using a motion encompassing method for managing tumor motion based on the use of an internal target volume (ITV), which is the standard method employed in our clinic. Clinically relevant dosimetric indices between gated and ITV-based plans were compared to evaluate benefits of gating with particular regard to normal lung tissue sparing.

## Methods

### Patients

Retrospective analysis was performed for 150 SABR plans that were each generated for individual tumors and were used to treat 128 medically inoperable NSCLC patients between 2010 and 2014. Patients with multiple tumors presented either with synchronous or metachronous primary tumors or both. All tumors were early-stage (T1-T3 (<5 cm)) to match criteria outlined in the report of Radiation Therapy Oncology Group (RTOG) 0915 [24]. Of 128 patients, 57 were male and 71 were female, and median patient age was 73 years (range: 32–96). Patients were immobilized using the BlueBAG BodyFIX® (Elekta, Stockholm, Sweden) immobilization system. Median tumor volume was 10.9 cm^3^ (range: 0.3–75.6 cm^3^). The distribution of tumors, sorted according to position relative to the proximal bronchial tree and according to lobe, is given in Table 1. Thirty-six tumors were located centrally, defined as being within 2 cm of the proximal bronchial tree [24] or mediastinal structures, and 114 lesions were located peripherally. Peripheral lesions were subdivided into “island” (*n* = 57) or lung wall-seated tumors (*n* = 57) using definitions given by Altman et al. [25]. Island tumors are enclosed by lung parenchyma, and lung wall-seated tumors abut the lung wall. Tumors were fairly evenly split between upper (*n* = 75) and lower (*n* = 65) lobes with 10 tumors located in the right middle lobe.

**Table 1:** Distribution of tumors evaluated in this paper. Tumors were sorted into two categories - according to position relative to the proximal bronchial tree and according to lobe

Tumor Location	
Peripheral - Lung Wall	57
Peripheral - Island	57
Central	36
Lower lobe	65
Middle lobe	10
Upper lobe	75
Total	150

### Margins

**Fig. 1:**
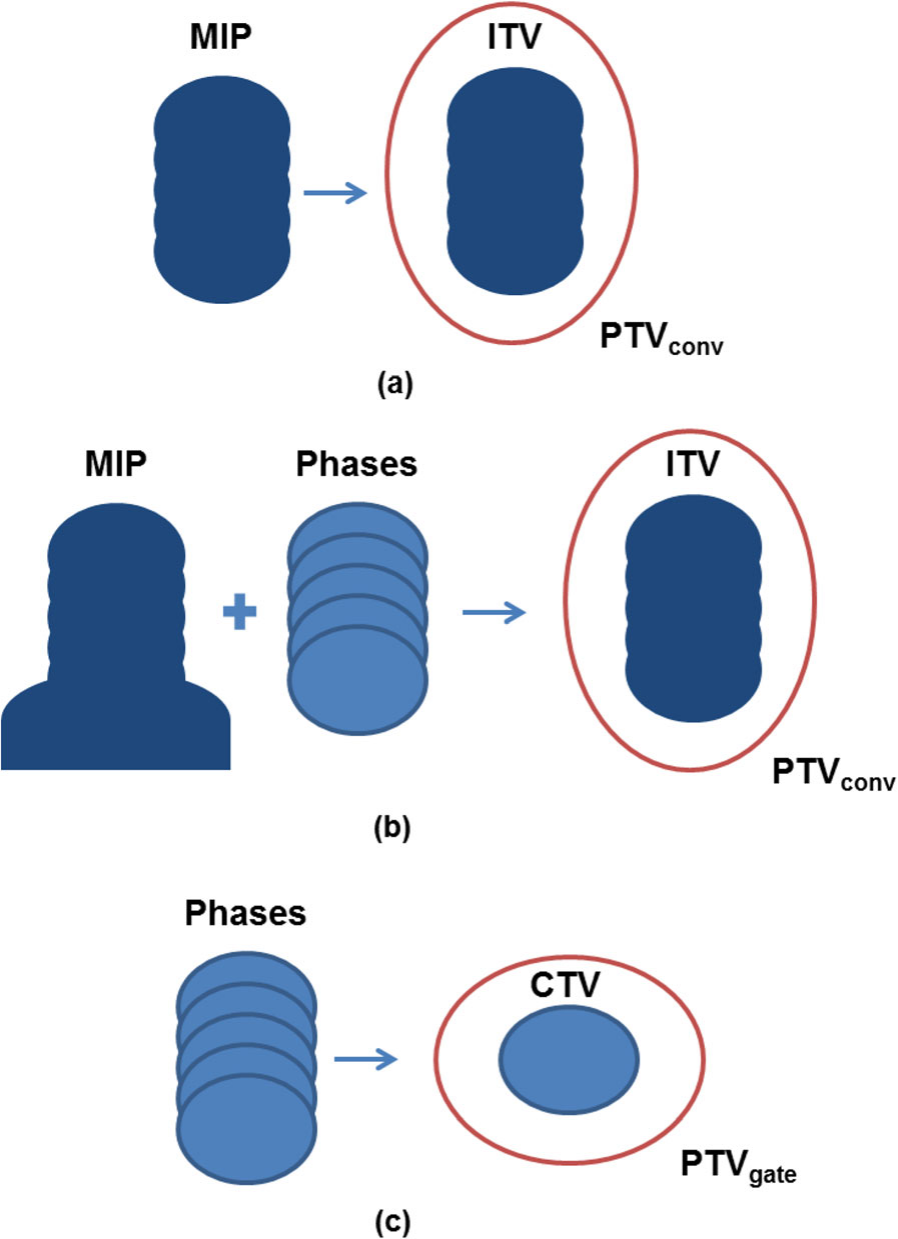
Gating and ITV-based volume definition. **a** For island tumors, ITVs were contoured using the maximum intensity projection of all reconstructed 4DCT phases and expanded isotropically to generate the PTV_conv_ (*red line*). **b** For tumors close to high intensity structures such as the liver, the structure overlaps the range of tumor motion and, therefore, contours of the individual phase images are used in addition to the maximum intensity projection to define the boundary of the ITV. **c** For gating, the 50 % phase of the 4DCT was chosen as the CTV and expanded isotropically to produce the gating PTV, PTV_gate_

Prior to planning, patient 4DCT scans were performed using a Philips Brilliance Big Bore CT simulator (Philips Health Care, Cleveland, OH), and four breathing phases were reconstructed using a phase-based binning method. For island tumors, the physician used the maximum intensity projection of the reconstructed phase images to define the tumor volume as shown in Fig. 1a. For tumors near or abutting high intensity structures that overlap the ITV in the maximum intensity projection such as the liver or chest wall, the relevant phase images are used to define the outline of the ITV near the high intensity structures as shown in Fig. 1b. To account for setup uncertainties, the ITV-based PTV, PTV_conv_, used for treatment was generated by isotropically expanding the ITV by 5 mm. For gated radiotherapy, one 4DCT phase is typically chosen to define the gating window. Free breathing gating about the end-exhalation (50 %) [26] phase is the most common choice for gating because of the smaller residual motion during end-exhalation and higher duty cycle. Therefore, the end-exhale phase was used to define the gating window for this study. As outlined in RTOG 0915, the gross tumor volume (GTV) was defined as the visible tumor within the CT pulmonary window, and the clinical target volume (CTV) was identical to the GTV [24]. The planning target volume (PTV) for gating, PTV_gate_, was determined by 5 mm, isotropic expansion of the CTV, as shown in Fig. 1c, where 2 mm defined a narrow gating window consistent with free-breathing gating using internal surrogates [27] and 3 mm accounted for setup uncertainties [26].

### Treatment planning

Patient plans for treatment were generated in the Eclipse® Treatment Planning System v11.0 (Varian Medical Systems, Palo Alto, CA) using an ITV approach by members of the physics and dosimetry team, who each had several years of planning SBRT cases. Of the 150 plans, 109 were intensity modulated radiation therapy (IMRT) plans, 36 were 3D conformal plans, and 5 were volume modulated arc therapy (VMAT) plans. The standard dose regimen at our institution was 12 Gy per fraction in 4 fractions, with optimization starting from normal tissue constraints consistent with the recommendations of RTOG protocol 0915 [24]. Optimization target and OAR objectives were made progressively more stringent in order to improve plan quality. To generate gated plans, the ITV-based treatment plan was copied, and the isocenter for the new gating plane was moved to the geometric center of the PTV_gate_ contour as determined in Eclipse. Treatment fields were then realigned to the new isocenter position. Optimization of the new PTV_gate_ plan was performed beginning with constraints derived from RTOG 0915 with more stringent lung constraints introduced later that were considered to be appropriate due to the use of the gating window. As with the conventional plans, the OAR and PTV objectives for the gated plans were made more stringent in a way that was unique to each patient in order to minimize OAR dose while maintaining plan quality. Dose was then recalculated based on the optimized fluence. The end-exhalation phase of the 4DCT was used as the treatment planning CT. Another commonly used high dose fractionation regimen explored in this study was 3 fractions of 18 Gy per fraction. To evaluate the 3 × 18 Gy schedule, original clinical treatment plans were modified with respect to fraction number and dose delivered per fraction. Modified plans were re-optimized using scaled target volume objectives and OAR constraints derived from the AAPM Task Group 101 [28] report with optimization objectives that were made more stringent just as with the 4 × 12 Gy schedule. A single physicist with experience in generating SBRT plans was responsible for both optimizing modified conventional plans and generating the gated plans. To establish plan quality independent of the planner, all plans were required to achieve standardized constraints with respect to plan conformity, target coverage, dose heterogeneity, and dose to OARs.

### Evaluation metrics

The main focus of the evaluation is lung toxicity. Analysis for lung toxicity will primarily be using mean lung dose (MLD) and lung volume percentage receiving doses of >20 Gy (V20). MLD [29] and V20 [24] have been shown to correlate well with incidence of radiation pneumonitis in conventional fractionation regimens. Trends between the total tumor motion (defined as the magnitude of the three-dimensional difference in position between the tumor’s center of mass at end-inhalation (0 % phase) and end-exhalation (50 % phase)) and the calculated difference between gated and ITV-based plans were investigated. For each methodology (ITV vs. Gated), lung was defined to be the total lung minus the PTV used for each method.

Additionally, we wanted to determine how the use of gating-based margins would affect doses calculated for other OARs. For each plan, only those OARs that were determined by the physician to be proximate enough to the tumor to receive significant dose were contoured and then included in the study (e.g. heart was contoured for central lesions, ribs for peripheral tumors). For this patient population, the most commonly contoured non-lung OARs were spinal cord (*n* = 133), heart (*n* = 80), esophagus (*n* = 70), and ribs (*n* = 53). Additionally, the bronchial tree and trachea were contoured and analyzed for thirteen centrally located tumors. Delivered doses as well as doses calculated using gating margins for both regimens were compared to recommended values of RTOG 0915 and AAPM TG101.

For all metrics, a two-tailed paired *t*-test was used to evaluate the significance of the results from using the two planning methodologies on the same patient population. A difference was considered to be statistically significant if the p-value was ≤0.05.

## Results

### Dosimetric evaluation

MLD and V20 results for the 3 × 18 Gy fractionation regimen are shown in Table 2. Only results for the 3 × 18 Gy regimen, where the largest differences were observed, are included in the table. Table 2 was divided into two halves with results sorted by position relative to the central region in the top half and results sorted by lobe in the bottom half. Dosimetric values were determined for both gated and ITV-based plans, with differences calculated by subtracting gated from ITV-based values. The range of dose values was reported in parentheses. For both gated and ITV-based plans, centrally located tumors showed the highest average absolute values for all metrics (e.g. gating central MLD = 4.05 ± 1.63 Gy vs. 2.94 ± 1.35 Gy for peripheral-lung wall and 3.35 ± 1.16 Gy for peripheral-island tumors) due to the increased volume of lung receiving radiation exposure for centrally located tumors. For all regions, the average dose reduction was small (<0.5 Gy and <0.7 % for MLD and V20, respectively) and statistically insignificant. The largest average dose reductions from gating plans relative to ITV plans were observed in the peripheral regions (particularly the lung wall). Average differences (ITV vs. Gated) were: 0.16 ± 0.64 Gy (lung wall) and 0.20 ± 1.17 % (lung wall) for MLD and V20, respectively, though none of the differences were statistically significant (*p* = 0.08 and *p* = 0.21, respectively). Interestingly, MLD and V20 differences between gating and ITV-based methods were essentially the same for both peripheral island tumors and peripheral lung wall tumors.

**Table 2:** Average MLD (Gy) and V20 (%) of gated and ITV plans as well as difference between the two (ITV-Gating) for 3 × 18 Gy fractionation. Ranges are given in parentheses. The V20 limit taken from RTOG 0236 is provided in brackets

	MLD (Gy)			V20 (%) [Limit: 10 %]		
	Gating	ITV	Difference	Gating	ITV	Difference
Peripheral-Lung Wall	2.94 ± 1.35 (0.69, 5.67)	3.10 ± 1.47 (0.83, 6.79)	0.16 ± 0.64 (−1.38, 2.82)	3.73 ± 2.24 (0.14, 8.89)	3.94 ± 2.24 (0.54, 8.32)	0.20 ± 1.17 (−4.54, 4.52)
Peripheral-Island	3.35 ± 1.16 (1.16, 6.05)	3.45 ± 1.18 (1.13, 6.16)	0.10 ± 0.56 (−1.72, 1.75)	4.40 ± 2.12 (0.95, 9.55)	4.59 ± 2.22 (1.27, 10.82)	0.20 ± 1.02 (−2.95, 3.01)
Central	4.05 ± 1.63 (1.06, 7.59)	4.15 ± 1.62 (1.19, 8.31)	0.10 ± 0.64 (−1.50, 2.52)	5.90 ± 3.61 (1.53, 14.96)	5.92 ± 3.37 (1.03, 16.93)	0.02 ± 1.30 (−3.54, 3.68)
LL	3.29 ± 1.43 (0.69, 7.59)	3.73 ± 1.56 (0.83, 8.31)	0.40 ± 0.55 (−0.59, 2.52)	4.27 ± 2.89 (0.14, 14.96)	4.93 ± 2.98 (0.58, 16.93)	0.66 ± 0.95 (−1.29, 3.68)
ML	3.10 ± 0.81 (2.02, 4.23)	3.33 ± 1.40 (1.79, 6.79)	0.23 ± 1.06 (−0.98, 2.82)	3.48 ± 1.14 (1.70, 5.03)	3.83 ± 2.01 (1.64, 8.32)	0.35 ± 1.72 (−1.71, 4.52)
UL	3.44 ± 1.44 (0.90, 7.11)	3.34 ± 1.41 (0.87, 6.60)	−0.11 ± 0.44 (−1.72, 1.12)	4.80 ± 2.64 (0.48, 14.00)	4.59 ± 2.43 (0.54, 12.45)	−0.24 ± 0.91 (−4.54, 3.05)

Sorted by lobe, the highest MLD values were seen in lower lobe tumors, but the highest V20 values were seen for upper lobe tumors. This was due to higher average motion and, therefore, larger PTVs seen for lower lobe tumors that resulted in a larger amount of healthy lung receiving a lower dose. At the same time, upper lobe tumors tended to have a smaller range of motion that resulted in small changes to PTV volume. With the PTV volume staying relatively constant combined with the effects of the smaller healthy lung volume in end-exhale images, the V20 values tended to be higher for upper lobe tumors. Consequently, dosimetric differences between gated and ITV-based plans were larger for lower lobe tumors than for upper lobe tumors, where average tumor motion was smaller (2.9 ± 3.4 mm (range: 0.1–17.0 mm) compared to 8.1 ± 6.1 mm (range: 0.1–28.1 mm) for lower lobe tumors). In fact, for upper lobe tumors, the average MLD and V20 were greater for the gating plans than for the ITV-based plans due to the effect of the smaller lung volume in the end-exhale image washing out any benefit from the slightly smaller target volume. The average tumor motion for middle lobe tumors (7.1 ± 6.7 mm) was less than for lower lobe tumors, resulting in slightly lower dose improvements for gating-based methods. Overall, while dosimetric reductions in the gating plans for any specific region were not statistically significant, the MLD reduction for the population as a whole was small (0.13 ± 0.60 Gy) but statistically significant (*p* = 0.012). No statistically significant differences were observed for V20. The same trends, but smaller in magnitude, in MLD and V20 results were observed for the 4 × 12 Gy regimen (as shown in Table 3).

**Table 3:** Average MLD (Gy) and V20 (%) of gated and ITV plans as well as difference between the two (ITV-Gating) for both fractionation regimens. Range of doses is given in parentheses. The V20 limit taken from RTOG 0236 is provided in brackets

	MLD (Gy)			V20 (%) [Limit: 10 %]		
	Gating	ITV	Difference	Gating	ITV	Difference
4 × 12 Gy	3.05 ± 1.27 (0.65, 7.08)	3.16 ± 1.34 (0.73, 7.52)	0.09 ± 0.57 (−2.14, 2.50)	3.88 ± 2.39 (0.46, 13.75)	3.96 ± 2.32 (0.37, 14.75)	0.08 ± 1.07 (−3.27, 4.09)
3 × 18 Gy	3.35 ± 1.40 (0.69, 7.59)	3.51 ± 1.48 (0.83, 8.31)	0.13 ± 0.60 (−1.72, 2.82)	4.49 ± 2.69 (0.14, 14.96)	4.69 ± 2.66 (0.54, 16.93)	0.19 ± 1.08 (−3.54, 4.52)

### Dose reduction as a function of tumor motion and volume

**Fig. 2:**
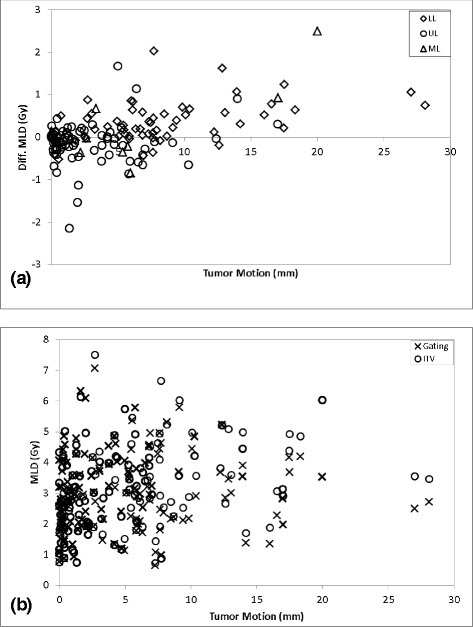
MLD vs. motion for 4 × 12 Gy regimen. Plot of difference in MLD between gating and ITV-based margins versus tumor motion for the **a** 4 × 12 Gy fractionation regimen. Data organized by lower lobe (*diamonds*), upper lobe (*circles*), and middle lobe (*triangles*). **b** Total MLD using both gating (*crosses*) and ITV-based (*circles*) margins plotted for each patient plan versus tumor motion are also provided

**Fig. 3:**
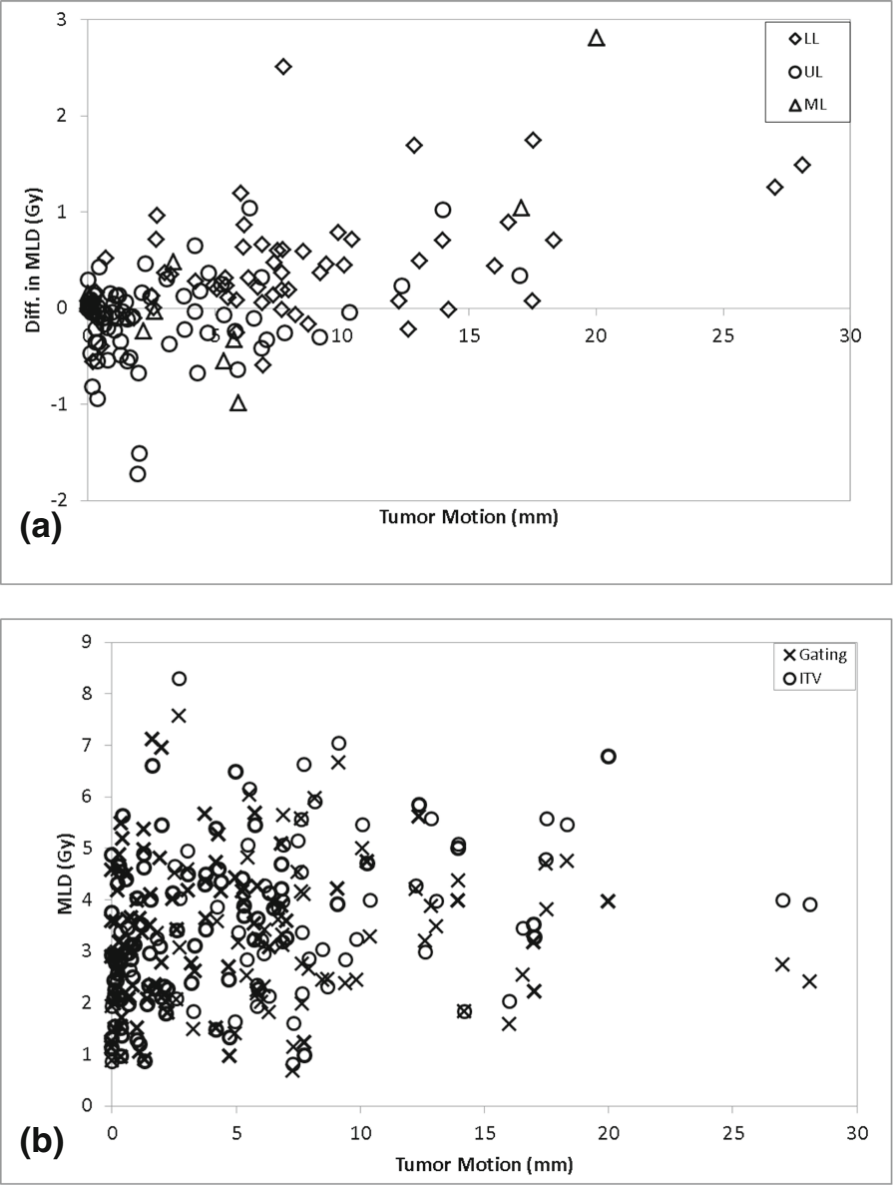
MLD vs. motion for 3 × 18 Gy regimen. Plot of difference in MLD between gating and ITV-based margins versus tumor motion for the **a** 3 × 18 Gy fractionation regimen. Data organized by lower lobe (*diamonds*), upper lobe (*circles*), and middle lobe (*triangles*). **b** Total MLD using both gating (*crosses*) and ITV-based (*circles*) margins plotted for each patient plan versus tumor motion are also provided

**Fig. 4:**
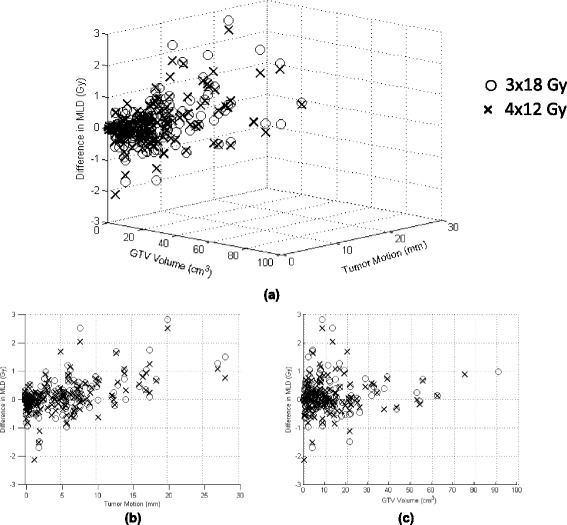
MLD as function of tumor motion and GTV volume. **a** 3D scatter plot of difference in MLD between gating and ITV-based margins as a function of both tumor motion and GTV volume for 3 × 18 Gy (*circles*) and 4 × 12 Gy (*crosses*) fractionation. **b** Magnitude of MLD increased with increasing tumor motion and **c** decreased with increasing tumor volume

**Fig. 5:**
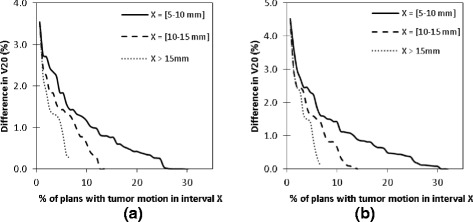
V20 difference as a function of tumor motion. Difference in V20 between ITV and gated margins used for patient plans with tumor motion in the 5–10 mm, 10–15 mm, and >15 mm intervals for **a** 4 × 12 Gy and **b** 3 × 18 Gy fractionation regimens

The relationship between tumor motion and MLD for both fractionation regimens is displayed in Figs. 2 and 3, respectively. In Figs. 2a and 3a, the reduction in MLD from implementing gating-based plans for 4 × 12 Gy and 3 × 18 Gy regimens, respectively, is plotted as a function of tumor motion with data organized by lobe. Upper lobe tumors (circles) were mainly clustered in the region of <5 mm motion while both lower lobe (diamonds) and middle lobe tumors (triangles) showed greater variability in the magnitude of motion. As can be seen in the figures, there was little difference between MLD values. Figures 2b and 3b display plots of absolute MLD calculated for both gated (crosses) and ITV-based (circles) plans for each patient, showing the trend of greater separation between the two methods as tumor motion increases. This can also be visualized in Fig. 4a, where the MLD reduction from using gating-based plans are plotted as a function of both tumor motion and volume for all 3 × 18 Gy (circles) and 4 × 12 Gy (crosses) patient plans. Just as in Figs. 2 and 3, Fig. 4b shows dose difference increasing as tumor motion increases. Overall, no benefit is seen in using the gating-based method for tumor motion less than 1.5 cm, with a small but increasing dose benefit past that point. Since tumor motion generally decreased as tumor volume increased, we expected that the dose reduction would be greatest for the smallest GTV. This was reflected in Fig. 4c where the percentage of plans with dose reduction exceeding 0.5 Gy decreased as GTV volume increased. To evaluate the magnitude of dosimetric effects for different ranges of motion, Fig. 5 displays absolute reduction in V20 from using gated plans with tumor motion in the intervals from 5 to 10 mm, 10–15 mm, and greater than 15 mm given as a percentage of the total number of patient plans. Approximately 29 % (*n* = 44) of plans had motion between 5 and 10 mm, 9 % (*n* = 13) between 10 and 15 mm, and 7 % (*n* = 11) greater than 15 mm. Within each group, absolute difference in V20 exceeded 2 % between gated and ITV-based plans for 9 % (*n* = 6), 13 % (*n* = 3), and 22 % (*n* = 2) of plans, respectively.

### Results for different fractionation regimens

Overall, differences between ITV and gated plans for MLD and V20 dose indices were found to be well below clinical tolerance values, considering radiation pneumonitis as the endpoint [30]. As observed in Table 3, this was borne out in the dosimetric results for both fractionation schedules. Figure 4 also showed that the magnitude of dosimetric difference between gated and ITV-based plans for the 4 × 12 Gy fractionation schedule followed the same patterns as for the 3 × 18 Gy fractionation schedule, but with a smaller magnitude. For the 4 × 12 Gy schedule (standard of care regimen in our department), the differences between ITV and gated treatment plans for all dose indices were small and deemed clinically irrelevant as lung doses already fell well below clinical tolerances and the calculated equivalent dose in 2 Gy fraction dose difference was rarely greater than 2 Gy (1 fraction) [31]. Moreover, no patient for any fractionation regimen approached clinically relevant V20 values (>10–15 % [24] of normal lung) based on RTOG 0915 recommendations [24].

### Dose to non-lung organs at risk

Mean dose to the spinal cord, heart, esophagus, and trachea remained within the recommended criteria given in RTOG 0915 [24], while the ribs and proximal bronchial tree infrequently exceeded the criteria due to unavoidable overlap with the PTV. OAR results as well as clinical endpoints are provided in Table 4. Additionally, the dose limits taken from RTOG 0915 and TG 101 are listed for the 4 × 12 Gy and 3 × 18 Gy regimens, respectively. Statistically significant differences between gating and ITV-based plans were only observed for the dose to 0.35 cc for the spine (*p* = 0.05) and dose to 5 cc for the esophagus (*p* = 0.038), both for 3 × 18 Gy regimens. Overall, average dose in gated plans tended to be slightly lower for the 4 × 12 Gy regimen but slightly higher for the 3 × 18 Gy regimen. While results were mixed with no overall benefit seen for non-lung OARs from using a gating-based method, less time was focused on optimizing dose to the OARs as was devoted to reducing dose to the healthy lungs for this study.

**Table 4:** Comparison of absolute dose between gating and ITV-based plans to (a) Spinal Cord, (b) Heart, (c) Ribs, (d) Esophagus, (e) Bronchial Tree, and (f) Trachea for each fractionation scheme. Clinical endpoints are listed for each organ as well as dose limits taken from RTOG 0915 and TG101 for 4 × 12 Gy and 3 × 18 Gy fractionation regimens, respectively

(a)	D_max_ to Volume = 0.35 cc (Gy)				D_max_ (Gy)			
Spinal Cord: Myelitis	Limit	Gating	ITV	Difference	Limit	Gating	ITV	Difference
4 × 12 Gy	20.8	9.03 ± 4.27 (2.2, 20.9)	9.30 ± 4.36 (1.9, 21.3)	0.49 ± 2.28 (−7.7, 10.5)	26	10.60 ± 4.93 (2.4, 25.1)	10.84 ± 5.00 (2.1, 25.7)	0.48 ± 2.79 (−10.1, 11.5)
3 × 18 Gy	18	9.88 ± 4.71 (2.3, 22.4)	10.28 ± 4.74 (1.8, 23.1)	0.55 ± 2.69 (−8.6, 10.4)	21.9	11.63 ± 5.49 (2.6, 28.0)	12.08 ± 5.44 (2.2, 26.5)	0.64 ± 3.24 (−11.4,10.9)
(b)	D_max_ to Volume = 15 cc (Gy)				D_max_ (Gy)			
Heart: Pericarditis	Limit	Gating	ITV	Difference	Limit	Gating	ITV	Difference
4 × 12 Gy	28	8.49 ± 8.08 (0.2, 35.8)	8.83 ± 4.40 (0.2, 31.7)	0.46 ± 2.11 (−7.7, 6.2)	34	15.93 ± 14.86 (0.2, 55.2)	16.06 ± 13.55 (0.2, 52.4)	0.37 ± 4.00 (−15.9, 12.7)
3 × 18 Gy	24	9.39 ± 8.80 (0.2, 36.2)	9.69 ± 8.01 (0.2, 35.4)	0.29 ± 2.59 (−10.4, 7.1)	30	17.95 ± 16.39 (0.2, 62.3)	17.81 ± 14.86 (0.2, 57.6)	−0.14 ± 5.13 (−16.8, 14.2)
(c)	D_max_ to Volume = 1 cc (Gy)				D_max_ (Gy)			
Ribs: Fracture	Limit	Gating	ITV	Difference	Limit	Gating	ITV	Difference
4 × 12 Gy	32	42.12 ± 9.97 (19.3, 55.1)	42.26 ± 9.70 (20.5, 57.0)	0.14 ± 4.19 (−7.3, 17.2)	40	49.83 ± 6.96 (21.3, 59.0)	49.38 ± 3.79 (26.4, 58.7)	−0.45 ± 3.12 (−11.0, 7.5)
3 × 18 Gy	28.8	47.80 ± 11.45 (21.4, 61.8)	46.65 ± 10.75 (23.1, 64.1)	−1.15 ± 4.88 (−11.1, 15.4)	36.9	55.98 ± 8.72 (23.6, 66.8)	54.97 ± 7.71 (29.9, 66.1)	−1.01 ± 3.61 (−10.8, 10.4)
(d)	D_max_ to Volume = 5 cc (Gy)				D_max_ (Gy)			
Esophagus: Stenosis	Limit	Gating	ITV	Difference	Limit	Gating	ITV	Difference
4 × 12 Gy	18.8	5.91 ± 4.83 (0.3, 21.5)	6.45 ± 4.68 (0.4, 17.8)	0.54 ± 1.95 (−6.3, 8.4)	30	11.96 ± 9.36 (0.6, 48.1)	12.48 ± 7.99 (0.6, 35.3)	0.53 ± 3.97 (−14.6, 16.3)
3 × 18 Gy	17.7	6.59 ± 5.35 (0.4, 24.2)	7.08 ± 5.10 (0.4, 19.8)	0.49 ± 2.26 (−7.6, 9.1)	25.2	13.55 ± 10.32 (0.7, 52.8)	13.85 ± 8.42 (0.7, 35.7)	0.30 ± 4.53 (−17.3, 13.8)
(e)	D_max_ to Volume = 4 cc (Gy)				D_max_ (Gy)			
Bronchial Tree: Stenosis	Limit	Gating	ITV	Difference	Limit	Gating	ITV	Difference
4 × 12 Gy	15.6	9.52 ± 9.46 (0.8, 34.8)	8.54 ± 7.30 (0.8, 24.3)	−0.99 ± 2.97 (−10.5, 1.5)	34.8	25.95 ± 16.63 (8.5, 54.4)	24.37 ± 13.92 (10.0, 52.0)	−1.58 ± 4.65 (−14.4, 2.4)
3 × 18 Gy	15	11.02 ± 10.86 (0.9, 38.6)	9.19 ± 7.53 (0.9, 25.4)	−1.83 ± 3.98 (−13.2, 1.9)	30	29.08 ± 18.76 (8.3, 58.7)	26.39 ± 15.23 (10.9, 58.5)	−2.69 ± 6.09 (−16.8, 3.9)
(f)	D_max_ to Volume = 4 cc (Gy)				D_max_ (Gy)			
Trachea: Stenosis	Limit	Gating	ITV	Difference	Limit	Gating	ITV	Difference
4 × 12 Gy	15.6	1.07 ± 1.79 (0.1, 4.3)	1.29 ± 1.89 (0.1, 4.6)	0.22 ± 0.31 (0.0, 0.7)	34.8	2.47 ± 2.96 (0.1, 6.20)	2.73 ± 2.89 (0.1, 6.3)	0.26 ± 0.44 (0.0, 1.0)
3 × 18 Gy	15	1.27 ± 2.16 (0.1, 5.1)	1.50 ± 2.27 (0.1, 5.5)	0.24 ± 0.33 (0.0, 0.7)	30	3.01 ± 3.72 (0.1, 8.1)	3.28 ± 3.66 (0.1, 8.3)	0.28 ± 0.45 (0.0, 1.1)

## Discussion

In this study, gated plans were retrospectively generated for a large patient cohort in order to perform dosimetric comparisons with original ITV-based SABR plans used for treatment. Results indicate that dosimetric differences in equivalent dose in 2 Gy fractions between gating and ITV-based SABR plans for either fractionation regimen did not approach clinical relevance as defined by Ten Haken et al. [31] where a difference in 4 Gy (2 fractions) could be considered clinically relevant. The results indicate that this threshold may possibly be reached only for a population with very large (>4 cm) tumor motion, particularly for the 3 × 18 Gy regimen. The dose values were derived using the linear quadratic model for radiobiological effect [32, 33]. Different groups have proposed alternative models (e.g. the universal survival curve [34]) for evaluating the effects of SABR-based treatments. Dosimetric differences between ITV and gated plans should be considered in combination with the absolute MLD or V20 values to determine whether they are clinically relevant, so that even a small reduction in dose to the lung can be important when the MLD or V20 values are close to the constraint. Conversely, a relatively large reduction in dose could be irrelevant if the absolute dose fell well within constraints. Of all 150 treatment plans, only one patient (at the 3 × 18 Gy regimen) had an MLD value reduced by nearly 3 Gy in the gated plan. This was for a slightly below average volume (8.6 cc), peripheral tumor in the right middle lobe that had large tumor motion (>2 cm). For this patient, ITV-based MLD was only slightly over 8 Gy. This case emphasizes that the greatest benefit would be seen in patients with large tumor volumes that have greater than 2 cm motion, which tend to be concentrated in the peripheral regions. Typically, the target volume must be large to result in a lung dose that approaches constraints. However, as target volume increased, the target motion tended to decrease, limiting the benefit of using this gating methodology. Additionally, the added complexity associated with gating and the significantly increased patient compliance and treatment time should be factored into determining the utility of implementing a gating-based method.

OAR doses in the gated plans generally showed variable results. Dose to trachea and esophagus easily met the criteria for both methods, while dose to the heart dropped somewhat in central regions, mainly due to the lower percentage of heart volume being irradiated in gating plans. Meanwhile, dose to the ribs in gated plans were slightly higher than those for ITV-based plans. This is understandable since those patients with ribs contoured were typically lung wall seated patients where movement of the tumor was small and the PTV overlapped with the ribs. Therefore, the volume differences between the PTVs on the ITV and gated plans were small. Interestingly, the bronchial tree also received a slightly higher average dose in gated plans so that no benefit was seen in gating for those OARS where plans often failed to meet dose constraints.

Several motion management techniques are used clinically including free-breathing gating [35], deep inspiration breath hold [20], and deep expiration breath hold [36]. While free-breathing with a gating window defined around the 4DCT end-exhalation phase is the most common clinical approach, due to better reproducibility [26], free-breathing with a gating window around the end-inhalation phase has possible advantages in terms of dose to the lung due to the larger lung volume during inspiration. However, gating on the end-inhale phase is also known to have significantly longer treatment times [37]. Additionally, deep expiration has been shown to be advantageous in terms of scan time and reproducibility [36], though it is quite limited for lung cancer patients, who often have other significant co-morbidities, such as COPD, making it difficult for them to undergo breath-hold treatment. Margins used in this paper are consistent with those used for free-breathing using an internal surrogate reported by Shirato et al. [27]. Berbeco [38] found that when considering gating, patient-specific evaluation is necessary to properly account for residual motion, which could exceed 6 mm.

## Conclusions

For clinical relevance, absolute delivered values for V20 and MLD must be taken into account to justify the added logistical requirements of gated radiotherapy. In this study, average MLD and V20 reduction was very small (~0.20 Gy and ~0.3 %, respectively) with only a few ITV-based plans where MLD reduction exceeded even 2 Gy. Few of these patients had total MLD or V20 values that approached dosimetric constraints. Differences were even smaller for the 4 × 12 Gy regimen. OAR results did not show meaningful improvement in gated plans. This is especially true for those sites (ribs and bronchial tree) most likely to exceed dose constraints where average dose to those sites increased compared to ITV-based plans. For tumors with motion of 2 cm or less, which is the range of tumors observed in this study, no significant dose reduction was observed by implementing a gating-based method.

## Abbreviations

AAPM: American Association of Physicists in Medicine
CTV: Clinical target volume
GTV: Gross tumor volume
IMRT: Intensity modulated radiation therapy
ITV: Internal target volume
MLD: Mean lung dose
NSCLC: Non small cell lung cancer
OAR: Organ at risk
PTV: Planning target volume
PTV_gate_: Planning target volume for gating
RTOG: Radiation Therapy Oncology Group
SABR: Stereotactic ablative body radiotherapy
V20: Percent volume receiving 20 Gy
VMAT: Volume modulated arc therapy
